# Aspergillus nidulans Septa Are Indispensable for Surviving Cell Wall Stress

**DOI:** 10.1128/spectrum.02063-21

**Published:** 2022-02-02

**Authors:** Ryland N. Spence, Walker Huso, Harley Edwards, Alexander Doan, Samantha Reese, Steven D. Harris, Ranjan Srivastava, Mark R. Marten

**Affiliations:** a Department of Chemical, Biochemical and Environmental Engineering, University of Maryland Baltimore County, Baltimore, Maryland, USA; b School of Biological Sciences, University of Nebraska, Lincoln, Nebraska, USA; c Department of Plant Pathology and Microbiology, Iowa State University, Ames, Iowa, USA; d Department of Entomology, Iowa State University, Ames, Iowa, USA; e Department of Chemical & Biomolecular Engineering, University of Connecticut, Storrs, Connecticut, USA; Westerdijk Fungal Biodiversity Institute

**Keywords:** *Aspergillus nidulans*, septation, fungal cell wall, antifungal

## Abstract

Septation in filamentous fungi is a normal part of development, which involves the formation of cross-hyphal bulkheads, typically containing pores, allowing cytoplasmic streaming between compartments. Based on previous findings regarding septa and cell wall stress, we hypothesized that septa are critical for survival during cell wall stress. To test this hypothesis, we used known Aspergillus nidulans septation-deficient mutants (Δ*sepH*, Δ*bud3*, Δ*bud4*, and Δ*rho4*) and six antifungal compounds. Three of these compounds (micafungin, Congo red, and calcofluor white) are known cell wall stressors which activate the cell wall integrity signaling pathway (CWIS), while the three others (cycloheximide, miconazole, and 2,3-butanedione monoxime) perturb specific cellular processes not explicitly related to the cell wall. Our results show that deficiencies in septation lead to fungi which are more susceptible to cell wall-perturbing compounds but are no more susceptible to other antifungal compounds than a control. This implies that septa play a critical role in surviving cell wall stress.

**IMPORTANCE** The ability to compartmentalize potentially lethal damage via septation appears to provide filamentous fungi with a facile means to tolerate diverse forms of stress. However, it remains unknown whether this mechanism is deployed in response to all forms of stress or is limited to specific perturbations. Our results support the latter possibility by showing that presence of septa promotes survival in response to cell wall damage but plays no apparent role in coping with other unrelated forms of stress. Given that cell wall damage is a primary effect caused by exposure to the echinocandin class of antifungal agents, our results emphasize the important role that septa might play in enabling resistance to these drugs. Accordingly, the inhibition of septum formation could conceivably represent an attractive approach to potentiating the effects of echinocandins and mitigating resistance in human fungal pathogens.

## OBSERVATION

Septation in filamentous fungi involves the construction of cross-wall bulkheads along the hyphae during normal growth (1), is analogous to animal cell cytokinesis (2), and is closely coordinated with the completion of mitosis (2). Septa are composed of materials similar to those found in the fungal cell wall (e.g., chitin, β-1,3-glucan, and a glucan-peptide-galactosamine layer [3]) and typically have a central pore (allowing the exchange of molecules/organelles between compartments) which can be plugged during stress to compartmentalize, and therefore mitigate, damage (4, 5). Previous studies in Aspergillus fumigatus (6) and Candida albicans (7) show that the presence of septa increases cell viability during exposure to cell wall-damaging compounds. Consistent with this, we and others have recently found increased septation as a response to micafungin (a cell wall-damaging echinocandin) exposure (8–10). Specifically, we found that cells treated with micafungin formed more septa per hyphal unit area compared to a control. In contrast, anecdotal observations in our lab have not revealed a similar increase in septation when fungi are exposed to other stress agents. These findings led us to hypothesize that septa are required for survival during various kinds of cell wall stress but may not be necessary for surviving other, non-wall-related, stresses.

To test this hypothesis, we used four previously described septation-deficient deletion mutants: Δ*sepH*, Δ*bud3*, Δ*bud4*, and Δ*rho4*. The Aspergillus nidulans SepH, Bud3, Bud4, and Rho4 proteins have all previously been shown to be required for the formation of septa through different cellular mechanisms (11–13). To elicit cellular stress, we used three antifungal compounds known to activate the cell wall integrity signaling (CWIS) pathway by inducing cell wall stress (14), including calcofluor white (15–17), Congo red (14, 15), and micafungin (18). We also tested three non-cell wall stressors, including 2,3-butanedione monoxime (19), cycloheximide (20), and miconazole (21).

We used the four septation-deficient deletion mutants effectively as “cellular sensors” to examine the importance of septa for the survival of fungi exposed to cell wall-related, and -unrelated, stressors. Our results imply that septa are required for survival in the three cell wall stressors tested but are dispensable for survival in the three non-cell wall stressors tested. These results support our hypothesis and demonstrate the potential utility of antifungal therapies which target both septation and the cell wall simultaneously.

To confirm that previously published deletion mutants demonstrated deficient septation during unstressed growth, we captured 30 images of each strain after 16 h growth in a complex medium (YGV, Fig. S1). While the control strain developed multiple septa, none of the deletion mutants contained any septa. This is consistent with previous findings (11, 12, 22) documenting the absence of septa in these mutants.

**FIG 1:**
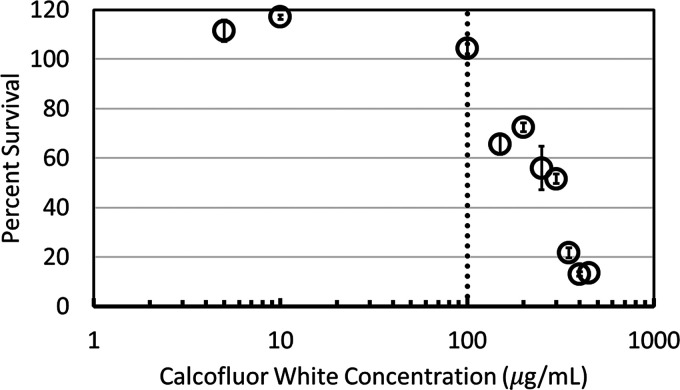
Dose-response curve for calcofluor white. One hundred fresh spores of the control strain were grown on (MAGV+UU) plates with increasing concentrations of calcofluor white. The critical concentration (dotted line) was determined to be 100 μg/mL; below this level, there was little impact on the cell survival (i.e., >80% survival). Three biological replicates were conducted at each concentration. The error bars represent the standard error.

To determine a suitable (i.e., “critical”) concentration of each stressor for testing septation-deficient mutants, we developed a dose-response curve for the control strain with each of the six stressors. Figure 1 shows the dose-response curve for calcofluor white; the others are shown in the supplemental material. Data exceeding 100% at low concentrations of calcofluor white are not statistically significant. The critical concentration is defined as the stressor concentration above which survival is <80%. We found the critical concentrations of calcofluor white, Congo red, and micafungin to be 100 μg/mL, 500 μg/mL, and 0.007 μg/mL, respectively, while the critical concentrations of cycloheximide, miconazole, and 2,3-butanedione monoxime were 200 μg/mL, 4.2 μg/mL, and 1.52 mg/mL, respectively.

**FIG 2:**
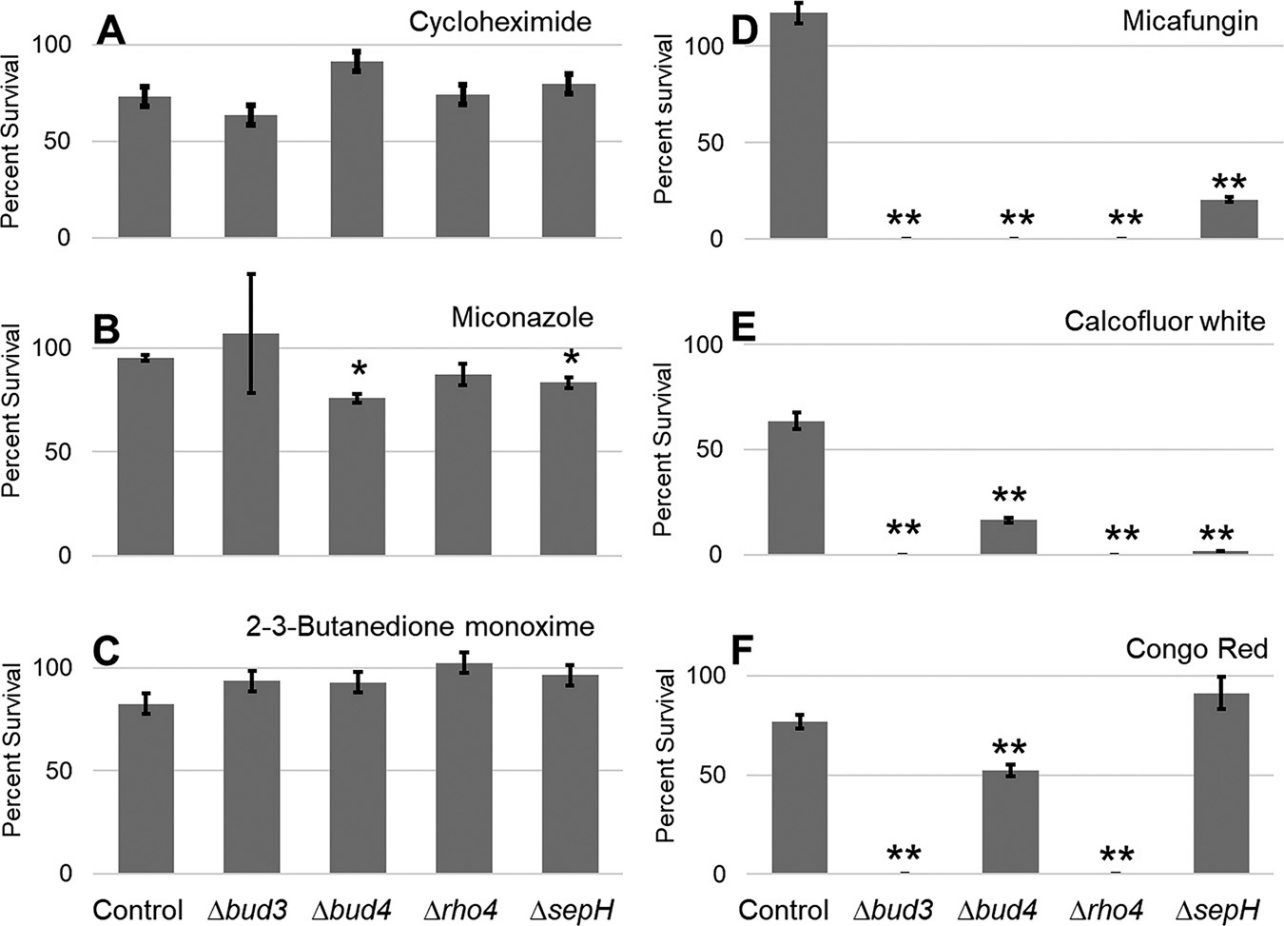
Susceptibility of A. nidulans to stressors. One hundred fresh spores of the control and septation-deficient mutants (Δ*bud3*, Δ*bud4*, Δ*rho4*, and Δ*sepH*) were grown on plates with, and without, a critical concentration of the indicated stress compounds. The number of colonies was counted on all plates, for three biological replicates. In the presence of non-cell wall stressors (A–C), nearly all septation-deficient mutants grew similarly to the control (*, *P < *0.05). In contrast, cell wall-related stressors (D–F) led to a significant reduction in the growth of the septation-deficient mutants (**, *P < *0.01).

We tested the control strain and each of the four septation-deficient strains (i.e., Δ*bud3*, Δ*bud4*, Δ*rho4*, and Δ*sepH*) with each of the six stressors at the determined critical concentrations. Figure 2 shows the results. For non-cell wall stressors (Fig. 2A to C), only two cases (i.e., Δ*bud4* and Δ*sepH* with miconazole) showed a significant decrease in survival. In contrast, with cell wall stressors (Fig. 2D to F), the septation-deficient mutants grew significantly less than the control in nearly all cases. The only exception was growth of the Δ*sepH* mutant in the presence of Congo red. While it is unclear why this mutant grew under these conditions, a possible explanation is that Congo red induced the activation of an alternate stress pathway able to compensate for the absence of SepH. Overall, however, our results are generally similar for all four mutants, implying that septa are required for survival of various wall stresses but dispensable for the three alternate stressors tested.

Our results agree with previous studies regarding stress from echinocandins (6, 8) but broaden the scope of previous findings in three important ways: First, we used four, unique, septation-deficient mutants, suggesting the general importance of septa as key factors in cellular response to wall stress. Second, we used three wall stressors, each with a different mechanism of action, broadening the scope of previous findings beyond echinocandins. Finally, using two different categories of stressors (i.e., cell wall and non-cell wall related) suggests a critical role for septa in responding to wall stress and the relative dispensability of septa in responding to the non-cell wall stresses.

Echinocandin resistance is a growing problem in the treatment of fungal infections, resulting in increased MICs (23). This work implies that antifungal therapies which simultaneously target septation and cell wall synthesis have the potential to be more effective than therapies targeting cell wall synthesis alone. While the current septation-inhibiting compounds are toxic at the required doses in humans (6), there is an opportunity to explore novel ways to inhibit septation, thus allowing us to combine septation inhibition with cell wall perturbation to create highly effective combination therapies.

To carry out the work described above, the control (A1405) and Δ*sepH* (22) strains were obtained from the FGSC (Fungal Genetics Stock Center). The deletion strains Δ*bud3*, Δ*rho4*, and Δ*bud4* have been described previously (11–13). To make the plates and liquid medium, stressors were added to MAGV and YGV, respectively (8), with 0.122% uridine and 0.122% uracil added (i.e., MAGV+UU; YGV+UU). The stressors included calcofluor white (Millipore Sigma), miconazole (Toronto Research Chemicals), Congo red (Millipore Sigma), micafungin (Myonex), 2,3-butanedione monoxime (Millipore Sigma), and cycloheximide (Fisher Scientific).

To assess the degree of septation, all strains were grown on coverslips at 28°C (24). After growing for 16 h, the slides were removed. At least 30 images were captured, with at least one mycelium per picture, with three biological replicates per strain. 

The “critical concentration” is defined as the stressor dose above which <80% viability was observed in the control strain; at higher doses, there was a visible decrease in survival. This is the highest dose that does not meaningfully affect the control; therefore, the impact on the deletion mutants at the same dose would be maximized. Deletions in a gene that is likely important in mediating the response to a stressor should show significantly decreased growth at this concentration. Critical concentrations were determined by creating dose-response curves for the control strain with each stressor.

To test the mutant susceptibility to stress, all strains were grown on agar plates with the stressors listed above at the determined critical concentrations. Fresh spores were harvested from MAGV+UU plates, diluted in sterile water to 1 spore/μL, and 100 μL of the spore suspension was spread onto both the control (MAGV+UU) and experimental (MAGV+UU+stressor) plates, with 3 biological replicates of each. The plates were grown at 28°C, and the number of colonies was counted after 3 days. The average number of colonies on the experimental plates was normalized to the average number of colonies on the control plates for each deletion mutant. Furthermore, the percent survival was calculated by dividing the average number of colonies on the experimental plates by the average number of colonies on the control plates. The statistical significance was determined using a Student’s *t* test to compare the average percent survival of each deletion mutant to the average percent survival of the control strain (*n* = 3). The results were considered significant when *P < *0.05 or *P < *0.01.

## References

[1] Walther A, Wendland J. 2003. Septation and cytokinesis in fungi. Fungal Genet Biol 40:187–196. doi:10.1016/j.fgb.2003.08.005. 14599886

[2] Balasubramanian MK, Bi E, Glotzer M. 2004. Comparative analysis of cytokinesis in budding yeast, fission yeast and animal cells. Curr Biol 14:R806–R818. doi:10.1016/j.cub.2004.09.022. 15380095

[3] Mourino-Perez RR, Riquelme M. 2013. Recent advances in septum biogenesis in Neurospora crassa. Adv Genet 83:99–134. doi:10.1016/B978-0-12-407675-4.00003-1. 23890213

[4] Beck J, Echtenacher B, Ebel F. 2013. Woronin bodies, their impact on stress resistance and virulence of the pathogenic mould Aspergillus fumigatus and their anchoring at the septal pore of filamentous Ascomycota. Mol Microbiol 89:857–871. doi:10.1111/mmi.12316. 23869404

[5] Tegelaar M, Bleichrodt R-J, Nitsche B, Ram AFJ, Wösten HAB. 2020. Subpopulations of hyphae secrete proteins or resist heat stress in Aspergillus oryzae colonies. Environ Microbiol 22:447–455. doi:10.1111/1462-2920.14863. 31736205 PMC6972715

[6] Dichtl K, Samantaray S, Aimanianda V, Zhu Z, Prévost M-C, Latgé J-P, Ebel F, Wagener J. 2015. Aspergillus fumigatus devoid of cell wall beta-1,3-glucan is viable, massively sheds galactomannan and is killed by septum formation inhibitors. Mol Microbiol 95:458–471. doi:10.1111/mmi.12877. 25425041

[7] Walker LA, Lenardon MD, Preechasuth K, Munro CA, Gow NAR. 2013. Cell wall stress induces alternative fungal cytokinesis and septation strategies. J Cell Sci 126:2668–2677. doi:10.1242/jcs.118885. 23606739 PMC3687699

[8] Chelius C, Huso W, Reese S, Doan A, Lincoln S, Lawson K, Tran B, Purohit R, Glaros T, Srivastava R, Harris SD, Marten MR. 2020. Dynamic transcriptomic and phosphoproteomic analysis during cell wall stress in Aspergillus nidulans. Mol Cell Proteomics 19:1310–1329. doi:10.1074/mcp.RA119.001769. 32430394 PMC8014999

[9] Souza ACO, Martin-Vicente A, Nywening AV, Ge W, Lowes DJ, Peters BM, Fortwendel JR. 2021. Loss of septation initiation network (SIN) kinases blocks tissue invasion and unlocks echinocandin cidal activity against Aspergillus fumigatus. PLoS Pathog 17:e1009806. doi:10.1371/journal.ppat.1009806. 34370772 PMC8376064

[10] Zhou X, Ye J, Zheng L, Jiang P, Lu L. 2019. A new identified suppressor of Cdc7p/SepH kinase, PomA, regulates fungal asexual reproduction via affecting phosphorylation of MAPK-HogA. PLoS Genet 15:e1008206. doi:10.1371/journal.pgen.1008206. 31194741 PMC6592577

[11] Si H, Rittenour WR, Xu K, Nicksarlian M, Calvo AM, Harris SD. 2012. Morphogenetic and developmental functions of the Aspergillus nidulans homologues of the yeast bud site selection proteins Bud4 and Axl2. Mol Microbiol 85:252–270. doi:10.1111/j.1365-2958.2012.08108.x. 22651396

[12] Si H, Justa-Schuch D, Seiler S, Harris SD. 2010. Regulation of septum formation by the Bud3-Rho4 GTPase module in Aspergillus nidulans. Genetics 185:165–176. doi:10.1534/genetics.110.114165. 20176976 PMC2870952

[13] Bruno KS, Morrell JL, Hamer JE, Staiger CJ. 2001. SEPH, a Cdc7p orthologue from Aspergillus nidulans, functions upstream of actin ring formation during cytokinesis. Mol Microbiol 42:3–12. doi:10.1046/j.1365-2958.2001.02605.x. 11679062

[14] Levin DE. 2005. Cell wall integrity signaling in Saccharomyces cerevisiae. Microbiol Mol Biol Rev 69:262–291. doi:10.1128/MMBR.69.2.262-291.2005. 15944456 PMC1197416

[15] Roncero C, Duran A. 1985. Effect of Calcofluor white and Congo red on fungal cell wall morphogenesis: in vivo activation of chitin polymerization. J Bacteriol 163:1180–1185. doi:10.1128/jb.163.3.1180-1185.1985. 3897187 PMC219256

[16] de Nobel H, Ruiz C, Martin H, Morris W, Brul S, Molina M, Klis FM. 2000. Cell wall perturbation in yeast results in dual phosphorylation of the Slt2/Mpk1 MAP kinase and in an Slt2-mediated increase in FKS2-lacZ expression, glucanase resistance and thermotolerance. Microbiology 146:2121–2132. doi:10.1099/00221287-146-9-2121. 10974100

[17] Pringle JR. 1991. Staining of bud scars and other cell wall chitin with calcofluor. Methods Enzymol 194:732–735. doi:10.1016/0076-6879(91)94055-h. 2005820

[18] Fujioka T, Mizutani O, Furukawa K, Sato N, Yoshimi A, Yamagata Y, Nakajima T, Abe K. 2007. MpkA-dependent and -independent cell wall integrity signaling in Aspergillus nidulans. Eukaryot Cell 6:1497–1510. doi:10.1128/EC.00281-06. 17601879 PMC1951132

[19] Rivera-Molina FE, González-Crespo S, Maldonado-De la Cruz Y, Ortiz-Betancourt JM, Rodríguez-Medina JR. 2006. 2,3-Butanedione monoxime increases sensitivity to nikkomycin Z in the budding yeast Saccharomyces cerevisiae. World J Microbiol Biotechnol 22:255–260. doi:10.1007/s11274-005-9028-x. 25382940 PMC4222539

[20] Schneider-Poetsch T, Ju J, Eyler DE, Dang Y, Bhat S, Merrick WC, Green R, Shen B, Liu JO. 2010. Inhibition of eukaryotic translation elongation by cycloheximide and lactimidomycin. Nat Chem Biol 6:209–217. doi:10.1038/nchembio.304. 20118940 PMC2831214

[21] Morita T, Nozawa Y. 1985. Effects of antifungal agents on ergosterol biosynthesis in Candida albicans and Trichophyton mentagrophytes: differential inhibitory sites of naphthiomate and miconazole. J Invest Dermatol 85:434–437. doi:10.1111/1523-1747.ep12277141. 3902987

[22] De Souza CP, Hashmi SB, Osmani AH, Andrews P, Ringelberg CS, Dunlap JC, Osmani SA. 2013. Functional analysis of the Aspergillus nidulans kinome. PLoS One 8:e58008. doi:10.1371/journal.pone.0058008. 23505451 PMC3591445

[23] Perlin DS. 2015. Echinocandin resistance in Candida. Clin Infect Dis 61 Suppl 6:S612–S617. doi:10.1093/cid/civ791. 26567278 PMC4643482

[24] Chelius CL, Ribeiro LFC, Huso W, Kumar J, Lincoln S, Tran B, Goo YA, Srivastava R, Harris SD, Marten MR. 2019. Phosphoproteomic and transcriptomic analyses reveal multiple functions for Aspergillus nidulans MpkA independent of cell wall stress. Fungal Genet Biol 125:1–12. doi:10.1016/j.fgb.2019.01.003. 30639305

