# Supplementary material for: Aspergillus nidulans Septa Are Indispensable for Surviving Cell Wall Stress
Source: Microbiol Spectr. 2022 Feb 2;10(1):e02063-21. doi: 10.1128/spectrum.02063-21 (PMC8809332; doi:10.1128/spectrum.02063-21)
Supplement: SUPPLEMENTAL FILE 1 — Supplemental material. Download SPECTRUM02063-21_Supp_1_seq2.pdf, PDF file, 0.3 MB [file spectrum02063-21_supp_1_seq2.pdf]

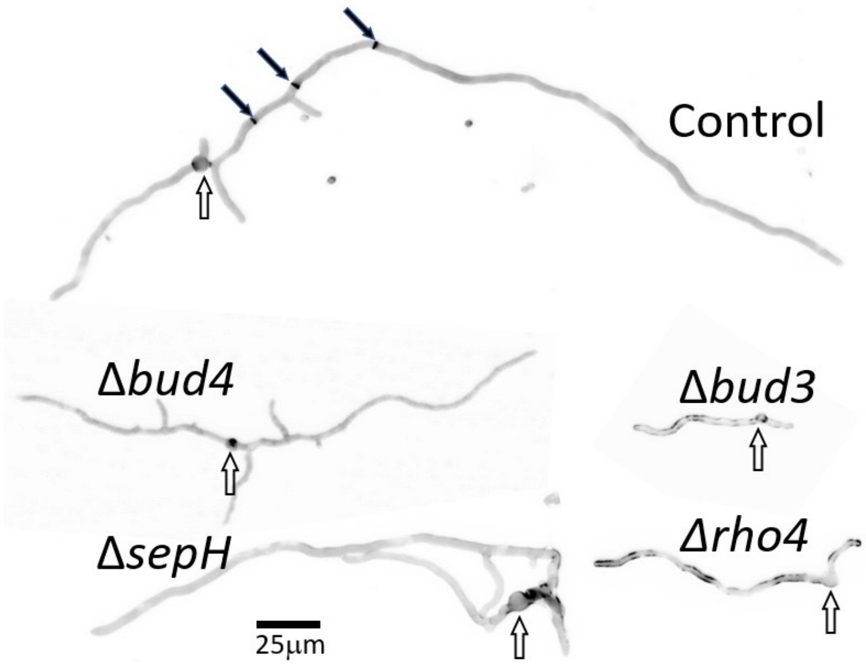

Figure S1**.** Representative fluorescent images of strains used in this work. All hyphae grown adhered to cover slips, in complex growth medium (YGV+UU), for 16h, and stained with Calcofluor white. Black arrows indicate septa, white arrows indicate spore centers. For each strain, 30 images were captured and hyphae shown are representative. Control strain shows normal *A. nidulans* septation. In contrast, none of the deletion strains contain any septa.

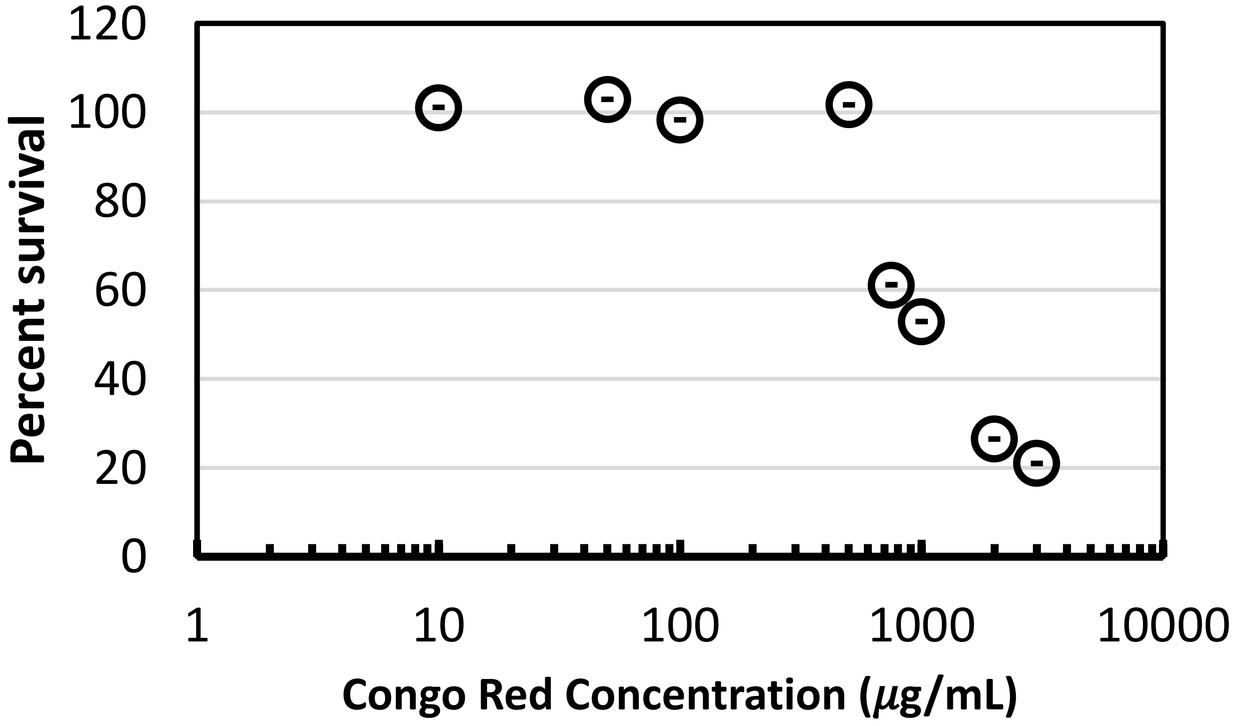

Figure S2. Dose response curve for congo red. One hundred fresh spores of the control strain were plated on MAGV+UU plates with increasing concentrations of congo red (0-3000 μg/mL) in order to find the "critical concentration" below which there is no impact on cell survival. Error bars represent standard error.

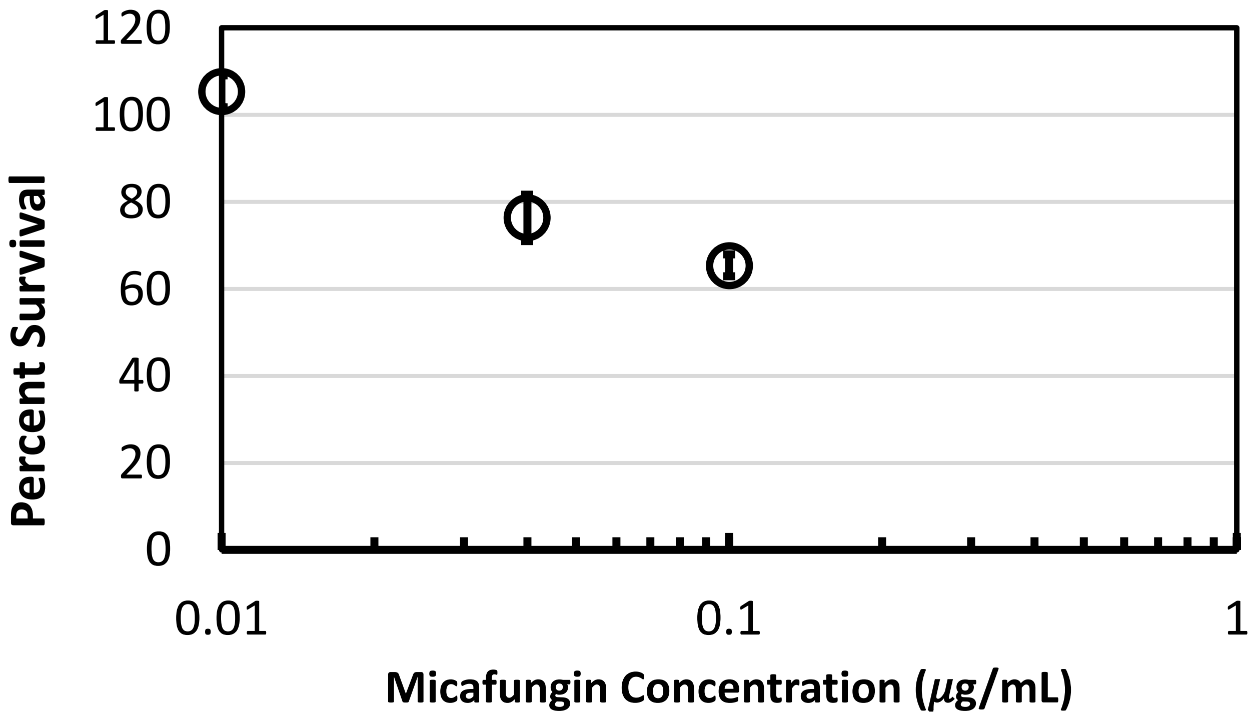

Figure S3. Dose response curve for micafungin. One hundred fresh spores of the control strain were plated on MAGV+UU plates with increasing concentrations of micafungin (0-0.1 μg/mL) in order to find the “critical concentration” below which there is no impact on cell survival. Error bars represent standard error.

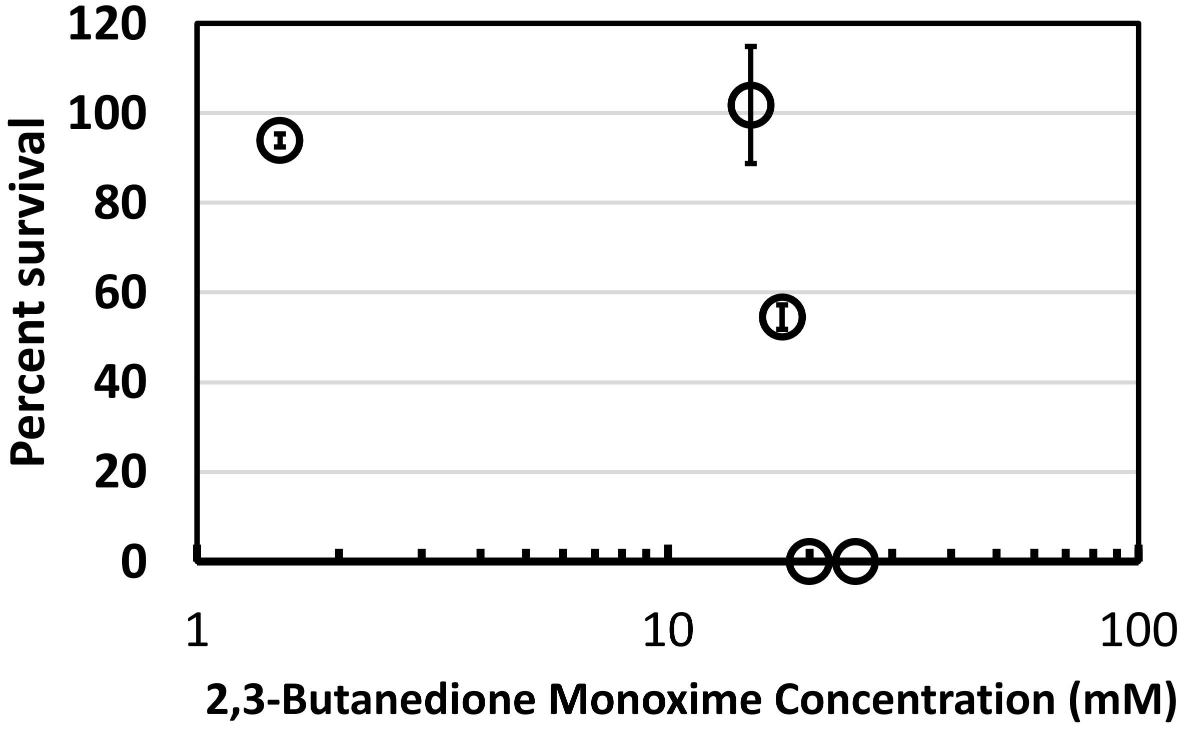

Figure S4. Dose response curve for 2,3-butanedione Monoxime. One hundred fresh spores of the control strain were plated on MAGV+UU plates with increasing concentrations of 2,3-butanedione monoxime (0-15 mM) in order to find the “critical concentration” below which there is no impact on cell survival. Error bars represent standard error.

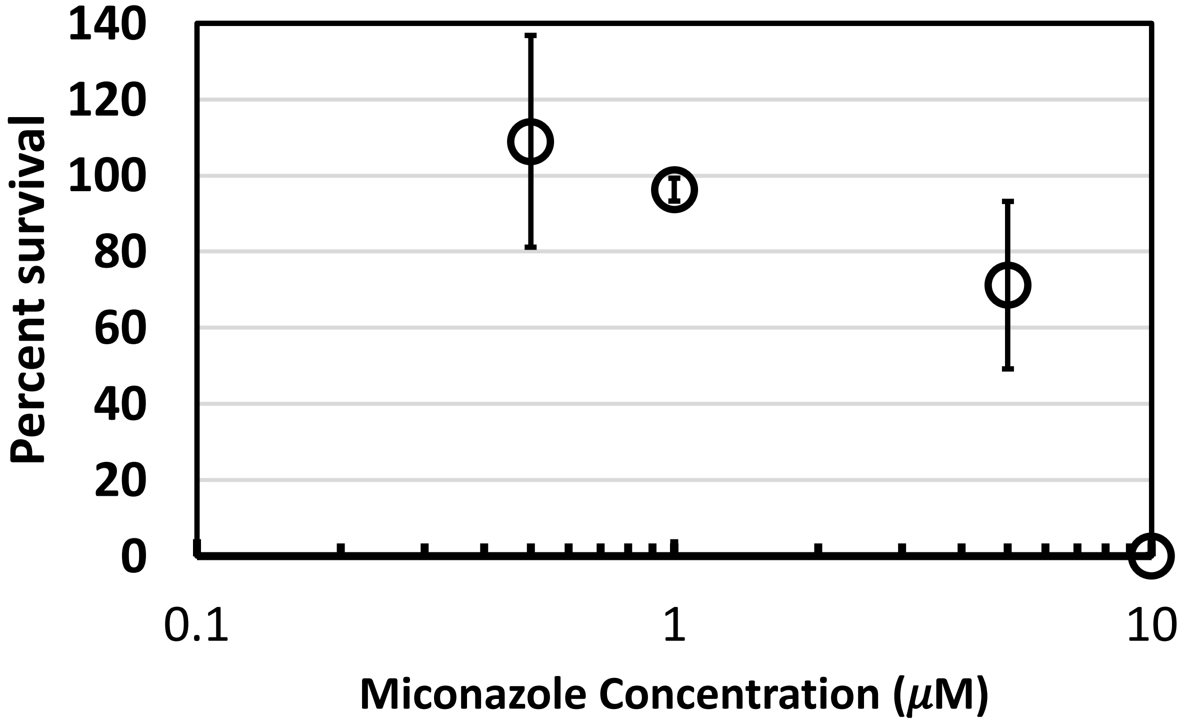

Figure S5. Dose response curve for miconazole. One hundred fresh spores of the control strain were plated on MAGV+UU plates with increasing concentrations of miconazole (0-10μM) in order to find the “critical concentration” below which there is no impact on cell survival. Error bars represent standard error.

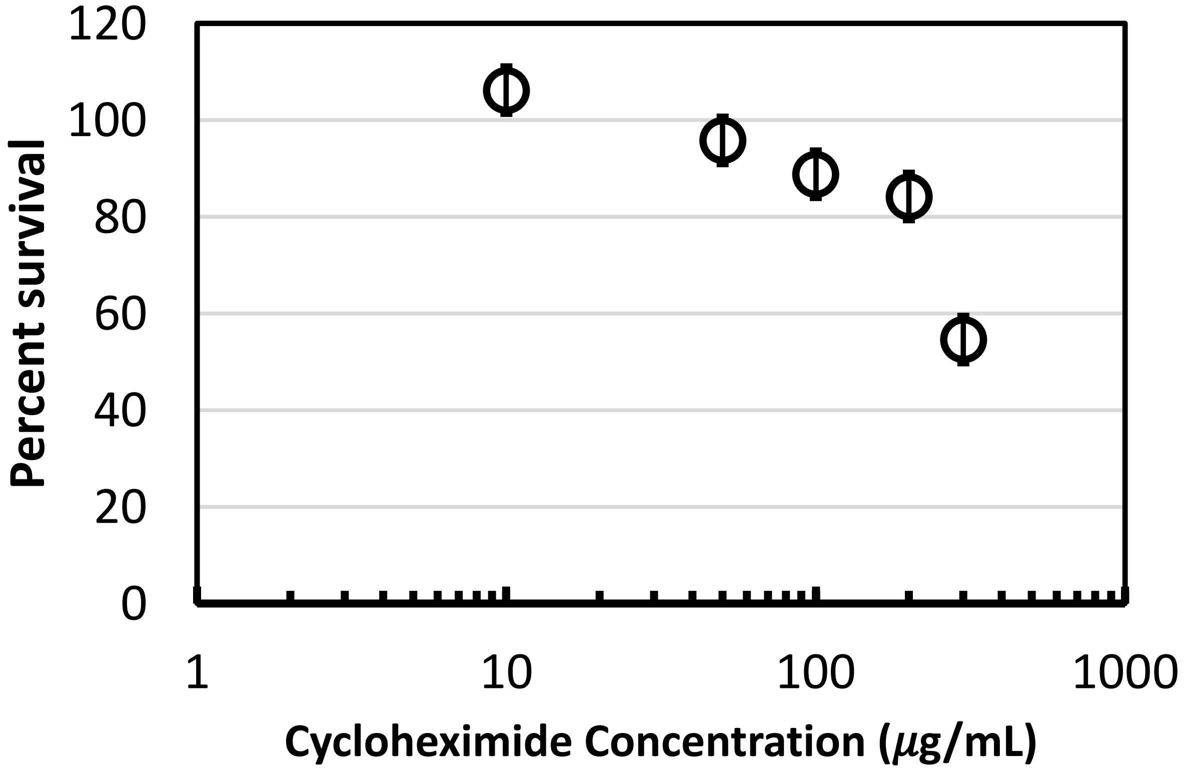

Figure S6. Dose response curve for cycloheximide. One hundred fresh spores of the control strain were plated on MAGV+UU plates with increasing concentrations of cycloheximide (0-300μg/mL) in order to find the "critical concentration" below which there is no impact on cell survival. Error bars represent standard error.
